# Serum Phosphorus Levels in Premature Infants Receiving a Donor Human Milk Derived Fortifier

**DOI:** 10.3390/nu7042562

**Published:** 2015-04-09

**Authors:** Katherine E. Chetta, Amy B. Hair, Keli M. Hawthorne, Steven A. Abrams

**Affiliations:** USDA/ARS Children’s Nutrition Research Center, Department of Pediatrics, Section of Neonatology, Baylor College of Medicine, Texas Children’s Hospital, Houston, TX 77030, USA; E-Mails: abhair@bcm.edu (A.B.H.); kelih@bcm.edu (K.M.H.); sabrams@bcm.edu (S.A.B.)

**Keywords:** neonate, phosphorus, hyperphosphatemia, human milk, exclusive human milk-based diet, human milk-derived fortifier, prematurity, creatinine, donor milk, very low birth weight

## Abstract

An elevated serum phosphorus (P) has been anecdotally described in premature infants receiving human milk fortified with donor human milk-derived fortifier (HMDF). No studies have prospectively investigated serum P in premature infants receiving this fortification strategy. In this single center prospective observational cohort study, extremely premature infants ≤1250 grams (g) birth weight (BW) were fed an exclusive human milk-based diet receiving HMDF and serum P levels were obtained. We evaluated 93 infants with a mean gestational age of 27.5 ± 2.0 weeks (Mean ± SD) and BW of 904 ± 178 g. Seventeen infants (18.3%) had at least one high serum P level with a mean serum P of 9.2 ± 1.1 mg/dL occurring at 19 ± 11 days of life. For all infants, the highest serum P was inversely correlated to the day of life of the infant (*p* < 0.001, *R*^2^ = 0.175) and positively correlated with energy density of HMDF (*p* = 0.035). Serum P was not significantly related to gender, BW, gestational age, or days to full feeds. We conclude that the incidence of hyperphosphatemia was mild and transient in this population. The risk decreased with infant age and was unrelated to gender, BW, or ethnicity.

## 1. Introduction

Human milk is the optimal source of nutrition for all infants, including preterm ones. The American Academy of Pediatrics (AAP) recommends that all infants <1500 g birth weight (BW) should receive human milk appropriately fortified [1]. An exclusive human-milk based diet is defined as mother’s own milk (or pasteurized donor milk when mother’s own milk is unavailable) fortified with a donor human milk-derived fortifier (HMDF), containing no preterm formula or cow milk protein-based fortifiers. This approach provides advantages over cow milk-containing products providing optimal growth rates, a lower rate of necrotizing enterocolitis, and decreased days of parenteral nutrition. Additionally, such an approach leads to lowered sepsis rates, decreased mortality, and shorter hospital stays [2,3,4,5,6,7].

Extremely premature infants are susceptible to growth failure, metabolic growth abnormalities, and poor neurodevelopmental outcomes [5,8,9,10,11]. Common metabolic derangements of extremely premature infants including hypocalcemia, hyperphosphatemia, and hypomagnesemia are usually secondary to immature hormone responses and renal dysfunction [8]. There are no published reports investigating the sequential early postnatal mineral serum chemistries of very low birth weight infants receiving an exclusive human milk-based diet with HMDF. This study aimed to evaluate how an exclusive human milk-based diet with HMDF can affect the risk of electrolyte abnormalities, specifically serum phosphorus.

There have been anecdotal reports of hyperphosphatemia associated with HMDF in extremely low birth weight infants and some health care providers may be limiting their use of this product because of this concern. Because of the potential benefits of using an exclusive human milk-based diet, but concern about phosphorus metabolism, we chose to evaluate the incidence of hyperphosphatemia, hypophosphatemia and hypocalcemia in all infants <1250 g BW receiving this diet.

## 2. Experimental Section

Extremely premature infants were consecutively followed in this single-center prospective observational cohort study from August 2010 to December 2011. Inclusion criteria were: premature infants <37 weeks gestation, BW <1250 g, admitted within 48 h of birth, receiving an exclusive human milk-based diet, and achievement of full enteral feedings by 4 weeks of age. Infants were excluded who died within the first week of life and those who had major congenital anomalies. Infants were followed from birth until discharge and data were prospectively collected for growth and nutrition using pre-study defined variables and definitions [3].

As approved by the Institutional Review Board of Baylor College of Medicine and Affiliated Hospitals, consent was waived for this observational study. Our primary outcome of this study was to evaluate the metabolic derangements in phosphorus levels for infants receiving an exclusive human milk-based diet with HMDF. Specifically, study outcomes included the number of phosphorus levels, mean phosphorus level drawn per infant, mean peak phosphorus level, percentage of infants with hyperphosphatemia (serum phosphorus > 8.0 mg/dL), percentage of infants with hypophosphatemia (serum phosphorus < 4.8 mg/dL), the incidence of hyperphosphatemia in relationship to serum creatinine, the incidence of hypocalcemia during hyperphosphatemia, and the day of life of peak phosphorus levels.

### 2.1. Standardized Feeding Protocol

Infants received a standardized feeding protocol which has been previously published by our group [3]. Human milk was fortified with HMDF Prolact +4, Prolact +6, Prolact +8, or Prolact +10 (Prolacta Bioscience, Industry, CA, USA) with final energy concentrations of 24 kcal/oz, 26 kcal/oz, 28 kcal/oz, and 30 kcal/oz, respectively, based on expected human milk energy concentration of 20 kcal/oz. Each fortifier adds 64 mg/dL of phosphorus and 122 mg/dL of calcium. This is comparable to most cow milk protein-based human milk fortifiers with phosphorus contents ranging from 26 mg/dL to 67 mg/dL and calcium contents ranging from 38 mg/dL to 138 mg/dL [2,12].

### 2.2. Data Collection

Serum phosphorus levels were collected per unit protocol three days after stopping parenteral nutrition and repeated one week later if serum phosphorus was >8.0 mg/dL. Further serum phosphorus values were collected at the discretion of the attending clinician. The peak phosphorus level (or single highest phosphorus level for one infant) was recorded along with the corresponding the day of life, degree of calorie fortification (energy density), and the number of days after discontinuation of parenteral nutrition. All infants with serum phosphorus levels >8.0 mg/dL were assessed with a serum creatinine, serum calcium, and serum alkaline phosphatase level within three days if available. Normal and low serum phosphorus levels in all infants were collected with corresponding day of life. Serum phosphorus levels were treated as one value if separated by less than 2 days.

Only serum phosphorus levels drawn during the use of the HMDF were used in this study. Of infants with multiple serum phosphorus levels on record, the highest level was considered the peak serum phosphorus level. Of infants with only one serum phosphorus level on record, that level was used.

### 2.3. Statistical Analyses

Relationships among variables were evaluated using general linear modeling in which hyperphosphatemia was the primary outcome. Regression analysis was used to compare relationships between peak serum phosphorus and day of life, energy density of HMDF, and days to achieve full feeds. Additionally, comparisons between serum phosphorus and gender, BW, gestational age, race, and >750 g or below 750 g BW were completed with univariate regression analysis. Statistical significance was defined as *p* < 0.05. A *t*-test was performed to compare the serum phosphorus and serum creatinine between an infant group within five days of discontinuation of parenteral nutrition and a group five days after discontinuation of parenteral nutrition. Analyses were completed using SPSS 22.0 (SPSS Inc., Chicago, IL, USA). All data are mean ± standard deviation unless otherwise noted.

## 3. Results

**Figure 1:**
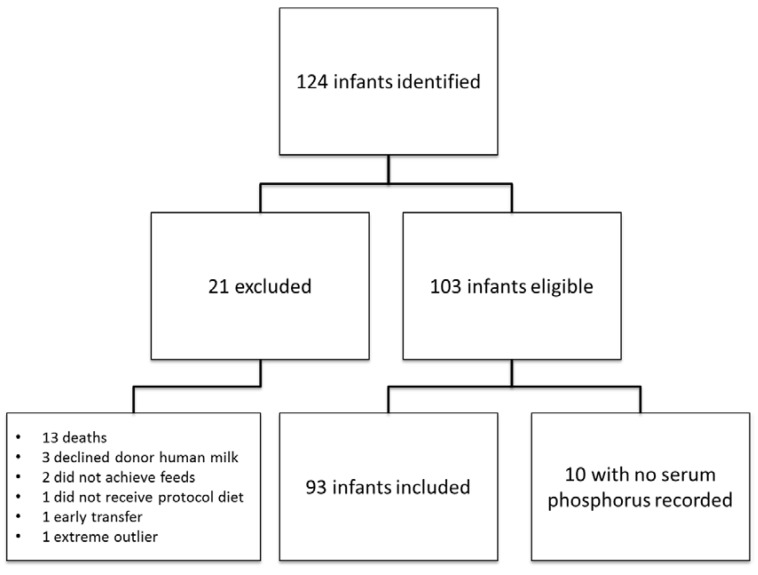
Infants receiving the exclusive human milk-based diet were prospectively followed.

Of 124 infants identified who initially met the inclusion criteria, 93 were included for the analysis. (Figure 1). The demographics and outcomes are shown in Table 1 and Table 2. In total, 356 serum phosphorus values were drawn among the 93 infants. On average, serum phosphorus levels were drawn 3.8 times per infant. Sixteen infants had only one serum phosphorus level checked, while 29 infants had serum phosphorus levels drawn over four times during their course (Table 3). Of 356 discrete levels during the use of HMDF, 291 (81%) serum phosphorus levels were within normal limits. The mean peak serum phosphorus was 7.2 ± 1.3 mg/dL. One infant was excluded for extreme hyperphosphatemia (serum phosphorus 17.8 mg/dL) at 48 days of life and was considered an outlier secondary to developing an incarcerated hernia at that time. Two infants also had hypocalcemia at the time of a high serum phosphorus level.

**Table 1:** Infant demographics and characteristics.

Demographics and Characteristics	Cohort *n* = 93
Birth weight, g	904 ± 178 *
Gestational age, weeks	27.5 ± 2.0
Male gender, *n* (%)	47 (51)
Race, % Black/White/Hispanic/Other	36, 25, 22, 10
Number of days to full 140 mL/kg/day feeds	16.0 ± 4.0
Inborn, *n* (%)	53 (57)
Antenatal steroids, *n* (%)	67 (72)

* Mean ± SD.

**Table 2:** Outcomes.

Outcomes	Cohort *n* = 93
Total serum phosphorus levels obtained	356
Mean number of serum phosphorus levels per infant	3.8 ± 2.5 *
Mean peak serum phosphorus level of all infants (mg/dL)	7.2 ± 1.3 *
Infants with high serum phosphorus (>8 mg/dL), *n* (%)	17 (18.3)
Total high serum phosphorus levels, *n* (%)	23 (7)
Infants with multiple high serum phosphorus levels, *n* (%)	5 (5)
Average high serum phosphorus level (mg/dL)	9.2 ± 1.1 *
Infants with low serum phosphorus (<4.8 mg/dL), *n* (%)	19 (20)
Total low serum phosphorus values (<4.8 mg/dL), *n* (%)	42 (13)
Infants with no serum phosphorus abnormalities, *n* (%)	64 (69)
Hypocalcemia occurring with high serum phosphorus level (serum calcium < 7.0 mg/dL or ionized calcium < 1.0 mmol/L), *n*	2

* Mean ± SD.

**Table 3:** Number of serum phosphorus levels recorded while on human milk-derived fortifier.

Number of Serum Phosphorus Levels Recorded Per Infant	*n* = 356
Only 1 level	16
2–3 levels	36
>4 levels	29
>6 levels	11

In the cohort, 17 infants (18.3%) had at least one laboratory result indicating hyperphosphatemia. Of these, five (5%) infants had more than one high level. Additionally, 19 (20%) infants had at least one episode of hypophosphatemia. Sixty-four (69%) of infants had all normal serum phosphorus levels. Of the infants with multiple high serum phosphorus levels, three infants had a serum creatinine over 1.0 mg/dL. Four of these infants received interventions (Table 4).

**Figure 2:**
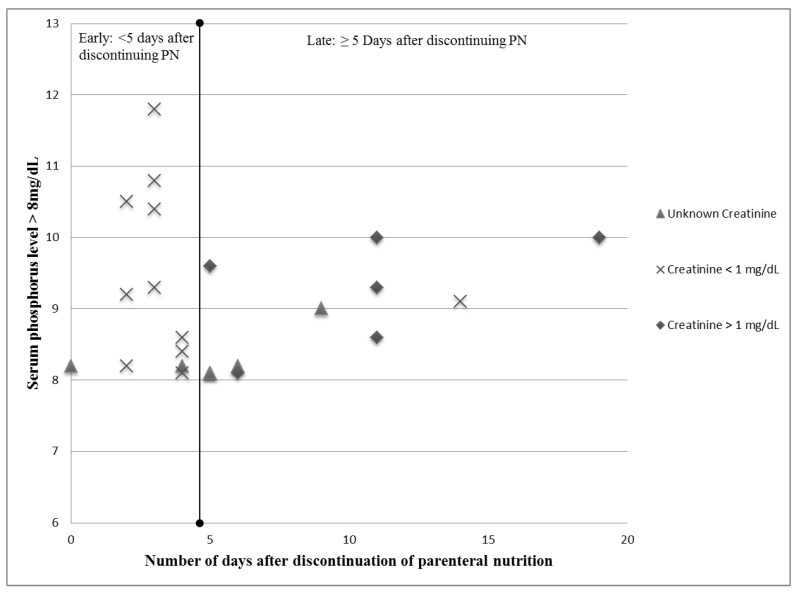
Comparison of creatinine levels in early *vs.* late hyperphosphatemia.

Ten (43%) of the high serum phosphorus levels were found to be within five days of transitioning off of parenteral nutrition. Eleven (45%) of the high serum phosphorus levels were associated with normal creatinine levels of <1 mg/dL, and of these 9 (81%) were within five days of the transition off of parenteral nutrition. Of the high serum phosphorus levels found five or more days after the transition off parenteral nutrition (12, 52%), half were associated with creatinine levels >1 mg/dL (Figure 2). Of the six high serum phosphorus levels associated with an elevated serum creatinine, the mean number of days from the transition off parenteral nutrition was 10.5 ± 5.0 days (Table 5). This is significantly different than the 11 high serum phosphorus levels associated with a normal creatinine found to be 4.9 ± 4.2 days from discontinuation of parenteral nutrition, *p* = 0.026.

**Table 4:** Outcomes of infants with multiple high serum phosphorus levels.

Infant	Serum Phosphorus	Intervention	Outcome	Serum Creatinine within 3 Days	Calorie Fortification (kcal/oz)
1	2 high values on day of life (DOL) 18 and 24 (both 8.2 mg/dL)	None	Serum phosphorus normalized on DOL 31	None	DOL 18: +4 DOL 24: +8
2	3 high values on DOL 13, 14, 19 (9.6, 8.1, and 9.3 mg/dL)	Treated for sepsis, found to have *Klebsiella* bacteremia	Serum phosphorus normalized on DOL 34	High creatinine 1.1 and 2.2 mg/dL, lowered DOL 24 (0.89 mg/dL)	DOL 13: +6 DOL 14: +8 DOL 19: +10
3	2 high values on DOL 12, 19 (8.4 and 10 mg/dL)	Worked up for sepsis. Cultures negative	Serum phosphorus normalized in subsequent checks on DOL 31, 52, 80	High creatinine 1.1 mg/dL lowered DOL 33 (0.55 mg/dL).	DOL 12: +6 DOL 19: +8 DOL 20: +10
4	2 high values on DOL 32, 45 (8.6 and 10.0 mg/dL)	Stopped HMDF for 1 day, worked up for sepsis	Serum phosphorus normalized in on DOL 50	High creatinine 1.3 mg/dL, normalized 0.4 mg/dL on DOL 114 (on formula)	DOL 32: +10 DOL 45: +10
5	2 high values on DOL 15, 23 (11.8 and 10.5 mg/dL)	HMDF held 1 day, DOL 16. Infant received IV calcium for hypocalcemia	Serum phosphorus normalized on DOL 20 and was normal on DOL 30 after resuming HDMF	Creatinine 0.74 and 0.91 mg/dL. Ionized calcium 0.84 mmol/L	DOL 15: +4 DOL 23: +10

**Table 5:** Comparison of serum creatinine and high serum phosphorus levels.

Serum Creatinine (± 3 Days of High Serum Phosphorus Level)	Number of High Serum Phosphorus Levels (*n* = 17)	Mean Days after Parenteral Nutrition Discontinued
High (≥1 mg/dL)	6	10.5 ± 5.0 *
Normal (<1 mg/dL)	11	4.9 ± 4.2 *

* Significant difference between groups *p* = 0.026.

**Figure 3:**
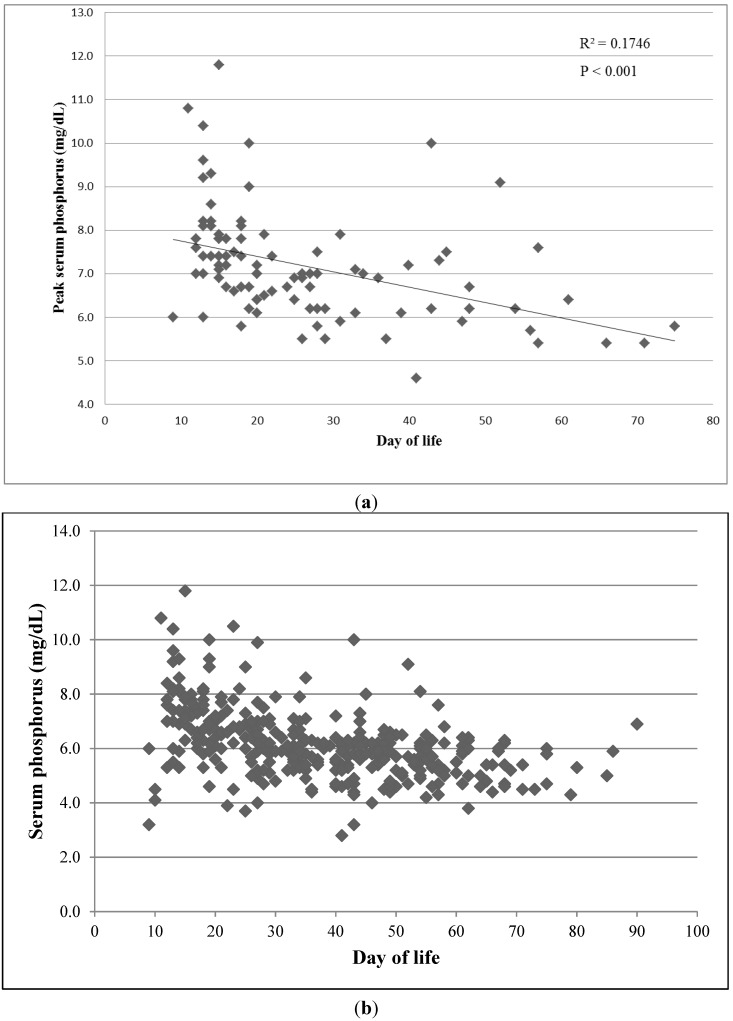
(**a**) Peak phosphorus level declines with day of life; (**b**) All serum phosphorus levels in 93 infants.

The serum phosphorus levels were closely associated with the age (day of life) of the infant (*p* < 0.001, *R*^2^ = 0.1746) (Figure 3a,b). The serum phosphorus levels were also significantly correlated to energy density concentration of HMDF used (*p* = 0.035). The number of days the infant needed to achieve full feeds (140 mL/kg/day) and any intrinsic characteristics of the infant including gender, BW, gestational age, race, or ≥750 g or < 750 g BW were not significantly associated with the risk of hyperphosphatemia.

Of 23 high serum phosphorus levels, 12 (52%) had serum calcium values drawn within three days. Ten of 12 levels were within normal limits. Two infants had laboratory evidence of hypocalcemia defined as < 7.0 mg/dL. One infant had a serum calcium level of 5.4 mg/dL and required a calcium infusion with the calcium level subsequently normalizing the next day. This infant had a creatinine level of 0.91 mg/dL and multiple high serum phosphorus levels. The other infant had a serum calcium level of 6.6 mg/dL which resolved three days later without supplementation. Alkaline phosphatase activity within 3 days was also recorded in 14 of the infants with high serum phosphorus levels and was found mildly elevated at 495 ± 215 mg/dL.

Very limited interventions were used in the infants with high serum phosphorus. The infants with multiple high serum phosphorus levels were more likely to receive interventions (Table 4) and often associated with high energy fortification (+8 and +10 kcal/oz). In four of the 17 infants with hyperphosphatemia, a decrease in daily feeding volume was made or the HMDF was stopped briefly (<3 days). These interventions coincided with a patent ductus arteriosus ligation and a clinically identified ileus associated with proven bloodstream infection for two infants. Most infants did not receive interventions, and all evidence of hyperphosphatemia resolved.

## 4. Discussion

Among 93 infants receiving an exclusive human milk-based diet, all serum phosphorus levels were analyzed with a focus on hyperphosphatemia. The average peak serum phosphorus level of infants receiving HMDF was <8.0 mg/dL. All infants received rapid advancement of feedings and increasingly high enteral phosphate loads.

Infants are susceptible to hypocalcemia and hypophosphatemia during prolonged parenteral nutrition and are often weaned to oral feedings rapidly as treatment [12]. Preterm infants fed unfortified mother’s milk early in life develop hypophosphatemia and elevated alkaline phosphatase levels leading to rickets [12,13,14,15,16]. Supplementation may protect against hypophosphatemia as premature infants have a limited body pool of phosphorus from their soft tissues risking demineralization leading to fractures [12]. Both hypophosphatemia and hyperphosphatemia occurred at similar rates in this study.

Seventeen (18%) infants in this study had hyperphosphatemia. The hyperphosphatemia was mild and transient occurring mostly early in life after the discontinuation of parenteral nutrition with few exceptions. In 17 infants with high serum phosphorus levels, 13 (76%) did not require intervention. The four interventions were minor dietary changes (slowing, stopping feeds or HMDF) for a short period of time. Two infants also had a patent ductus arteriosus ligation and clinically identified ileus at the time of intervention.

Hyperphosphatemia largely occurred within five days from the discontinuation of parenteral nutrition, at approximately 10–15 days of life. The risk of hyperphosphatemia decreased as the infant aged. Several mechanisms could contribute to early peak serum phosphorus levels including increasing feeding volumes, high absorption (bioavailability) of the phosphorus loads and suppression of parathyroid function by calcium in parenteral nutrition [12]. Parathyroid hormone surges are smaller in very low birth weight infants but function improves with postnatal age as nephrogenesis continues through 34 weeks post-gestational age [8,17].

Hyperphosphatemia occurring more than five days after the discontinuation of parenteral nutrition (late) was often associated with serum creatinine ≥1 mg/dL, possibly signifying renal dysfunction and an inability to excrete ingested phosphorus [17,18]. Our study also found that the higher energy density of HMDF was significantly associated with hyperphosphatemia (*p* = 0.025). Feeds were highly fortified (+8 kcal/oz or +10 kcal/oz) after evidencing poor weight gain on full (140–150 mL/kg/day) feeds. Poor weight gain is an independent marker of underlying renal dysfunction [18]. Renal function should be investigated in infants with late hyperphosphatemia. Multiple high serum phosphorus levels were also a risk factor for intervention. One infant received a calcium infusion secondary to hypocalcemia during an episode of hyperphosphatemia. Alkaline phosphatase activity was mildly high on average, however it is a not a highly specific marker for bone mineral defects in premature infants [12].

A limitation of this observational study was the non-randomized nature of this cohort. The prospective nature of this study was its strength, investigating a large cohort of premature infants that were only using an exclusive human milk-based diet, while analyzing serum phosphorus as an outcome.

## 5. Conclusions

An exclusive human milk-based diet provides phosphorus, supporting bone growth safely. Hyperphosphatemia was seen early in life with interventions being minor. Hypophosphatemia occurred with a similar frequency in this cohort. The risk of high serum phosphorus decreased with infant age unrelated to gender, BW, or ethnicity. An interesting point of this study was the correlation of high creatinine and late hyperphosphatemia. A clinician should be sensitive to serum phosphorus as a herald to renal dysfunction in these infants. When interpreting these interventions, recall that infants had comorbid renal dysfunction, sepsis, impending surgeries or a patent ductus arteriosus and had enteral feeds discontinued for those reasons rather than to treat hyperphosphatemia. Special consideration should be paid to electrolyte abnormalities when infants transition off of parenteral nutrition or demonstrate multiple high serum phosphorus levels as underlying renal dysfunction or rarely hypocalcemia may also be present. Monitoring serum phosphorus to assure a normalizing trend for this subset of premature infants receiving HMDF is supported.
